# Remodelling of the bone marrow vasculature induced by venetoclax and azacitidine damage

**DOI:** 10.1182/blood.2025030055

**Published:** 2026-06-11

**Authors:** Steven Ngo, Giuseppe D’Agostino, Despoina Papazoglou, Fatihah Mohamad Nor, Katja Finsterbusch, Khadidja Habel, Alessandra Ferrelli, Fernando Anjos-Afonso, Dominique Bonnet

**Affiliations:** 1 Haematopoietic Stem Cell Laboratory, The Francis Crick Institute, London, United Kingdom; 2 Plasticell Ltd, Stevenage Bioscience Catalyst, Stevenage, UK; 3 Immunoregulatory laboratory, The Francis Crick Institute, London, United Kingdom

## Abstract

The Bcl2 inhibitor venetoclax in combination with the hypomethylating agents azacitidine (ven/aza) has become increasingly utilized clinically for the treatment of many hematological malignancies. Whilst its effects on malignant cells have been extensively studied, its impact to the surrounding bone marrow microenvironment (BME) remains unexplored. In this study, we report that ven/aza therapy causes significant damage to the BME of mice. Comparatively high Bcl2 expression in the sinusoidal endothelial cell compartment (SEC) amongst all stromal subtypes, results in high sensitivity to ven/aza treatment, causing selective depletion of SECs and breakdown in cell-cell communication pathways in the endothelial cell (EC) network, leading to vascular leakiness in the BM. Furthermore, our detailed transcriptomic and imaging studies reveals significant downregulation of essential adhesion molecules in residual SECs, leading to significant defects in human hematopoietic stem/progenitor cell (HSPC) homing and engraftment of hematopoietic stem cells (HSCs) after ven/aza treatment. To conclude, our study showcases that maintaining SEC integrity in response to ven/aza therapy may play a key factor in achieving effective engraftment of donor derived HSCs.

## Introduction

The bone marrow microenvironment (BME) plays a vital role in the regulation of hematopoiesis, with recent advancements in single-cell sequencing unveiling the increasing cellular heterogeneity of the bone marrow microenvironment (BME) (1–4). The functional potential of each stromal cell subtype has yet to be fully elucidated, however this complex network of niche compartments collectively plays an integral role in maintaining hematopoietic stem cell (HSC) stemness (5). The vascular niche alone provides a rich source of key cytokines such as stem cell factor (SCF), CXC motif chemokine ligand 12 (CXCL12) and Notch ligands to promote HSC stemness and quiescence (6). Hence, disruption to the vasculature could lead to impaired HSC homing, premature exhaustion of HSCs and dysregulated hematopoiesis. Venetoclax is a clinically available Bcl2 inhibitor increasingly used to treat patients with hematological malignancies such as multiple myeloma (MM) and acute myeloid leukemia (AML) (7–9). In combination with hypomethylating agents such as azacitidine, venetoclax is currently used as a frontline therapy for high-risk AML patients and has significantly improved overall survival of such patients (7–9). Despite its efficacy, venetoclax and azacitidine (ven/aza) is not curative, and patients treated with the combinational therapy often require hematopoietic stem cell transplantation (HSCT) to achieve long-term survival (10, 11). Whilst molecular factors such as human leukocyte antigen (HLA) matching play a major role in determining effective engraftment, the ‘health’ of the BME itself prior to transplantation likely plays critical determinant for the success of donor-cell engraftment. Given the importance of the BME in establishing effective hematopoiesis, this study aims to characterize the impact ven/aza has on the BME and the potential implications this may have on HSCT. We hypothesize that venetoclax-related toxicity to the BME will in turn significantly compromise donor cell engraftment and effective BM reconstitution.

## Materials and Methods

### Animal handling and HSC homing/engraftment assays

NOD/SCID-IL2rγ^−/−^IL-3/GM/SCF (NSG-SGM3) mice were originally obtained from Leonard Shultz (The Jackson Laboratory) and crossed with NOD/SCID-IL2rγ^−/−^Tyr^+^Kit W41J (NBSGW) for 6 generations to create NBSGW-S mice. NBSGW mice were purchased from the Jackson Laboratory. C57BL6 mice were obtained from the communal colony at the Francis Crick Institute. All strains of mice were bred at the Francis Crick Institute Biological Resource facility. All animal experiments were performed under the U.K Home Office project license (70/8904) in accordance with The Francis Crick Institute animal ethics committee guidance and following the ARRIVE guideline. Male and female mice aged between 12-20 weeks, were used in these experiments. For HSCT homing assays, 70,000 CD34^+^ cells from umbilical cord blood (UCB) were injected intravenously and mice were culled 48h post injection. For HSCT engraftment assays 50,000 UCB CD34+ cells were injected intravenously and culled after 9-10 weeks. For secondary transplantation, 3,000 CD45^+^CD34+ CD38^-^ HSPCs were isolated from primary recipient mice and transplanted into NBSGW mice. The mice were then culled 12 weeks post transplantation to assess engraftment.

### Cell processing

Umbilical Cord Blood (UCB) was obtained after informed consent, in accordance with the Declaration of Helsinki from the Anthony Nolan Cell therapy service. Unless stated otherwise, three or more samples were pooled for each experiment and MNCs were obtained by density centrifugation using Ficoll-Paque. Human CD34^+^ cells from UCB were enriched using the EasySep™ Human CD34^+^ Selection Kit II (Stem Cell Technologies) according to the manufacturer’s protocol.

### Sample preparation and immunostaining for flow cytometry and FACs sorting

For isolation of bone ECs, the femurs, tibia and iliac crests were collected and flushed via centrifugation (2500 g for 1 min at room temperature). Please find details of enzymatic digestion in Supplementary Materials and Methods. The following antibodies were used for immunophenotyping of mouse ECs and FACs sorting for scRNAseq: anti-mouse CD45 BUV395 (clone 30-F11, BD Horizon), Ter119 PerCP-Cy5.5 (clone TER-119, eBioscience), CD71 PerCP-Cy5.5 (clone RI7217, Biolegend), Sca-1 BV421 (clone D7, Biolegend), ICAM1 (clone YN1/1.7.4,abcam), CD31 PE-Cy7 (clone 390, eBioscience), Lyve1 (clone ALY7, eBioscience), CD140a (clone APA5, Biolegend), CD62E (clone 10E9.6, BD Pharmingen), CD62P (RB40.34, BD OptiBuild). For annexin-V staining, isolated cells from the bone marrow were initially immunostained with the markers mentioned above, then stained with 5 μl of annexin-V reagent (640906, Biolegend) for 15 minutes at room temperature. Propidium Iodide (51-66211E, BD Biosciences) was used as a viability dye. In the case of fluorescence-activated cell sorting (FACs) for scRNAseq, cells were resuspended in PBS with 5% of FBS and sorted using the FACs Aria III BD with a 100 μm nozzle. For identification of transplanted HSCs, the following panel was used: CD34 FITC (clone 581, BD Pharmingen), CD38 APC-eFluor780 (clone HIT2, BD Pharmingen), CD45RA PE-Cy7 (clone HI100, eBioscience), CD135 A647 (clone 4G8, BD Pharmingen), CD90 PerCP-Cy5.5 (clone 43A3, Biolegend), CD201 PE (clone REA337, Miltenyi). DAPI was used as a viability dye. For staining of the BM vasculature using VE-CAD, mice were intravenously injected with VE-CAD antibody (BV13; Biolegend) 10 minutes prior to culling.

### Immunostaining for imaging

Immunofluorescent images were performed on 8 μm thick sections using a PhenoCycler Fusion 2.0. at 20X magnification (Akoya Biosciences). The antibodies used for immunostaining include: Endomucin (clone: V.7C7 eBioscience), Sca1 (clone: D7 Biolegend) and CD31 (clone MEC13, Akoya Biosciences). Endomucin and Sca-1 antibodies were custom conjugated to DNA barcodes using the Akoya Antibody Conjugation Kit (7000009, Akoya). Please find details of immunostaining preparation in Supplementary Materials and Methods.

### Single cell RNA-seq analysis

A detailed description of materials and methods is available as a Supplementary note. Briefly, reads were aligned to the mouse genome reference and quantified using CellRanger v. 7.1.0 using demultiplexing. Counts assigned to cells and experimental samples (vehicle, ven/aza, recovery) were imported in R as SingleCellExperiment objects and QC and filtering was carried out using standard indications from Bioconductor workflows (removing cells with too few total reads/genes detected, and too high percentage of mitochondrial and Malat1 expression). *SingleR* was used for reference-based annotation using a publicly available murine bone marrow stromal dataset (1) which led to the identification and removal of immune cells from our data.

Counts were normalized in a batch-aware function using functions from *scran* and *batchelor*, then highly variable genes were determined using a mean-variance trend fit and the distance from the fit as a measure of biological variance. 2082 highly variable genes were used as input for Principal Component Analysis (PCA) dimensionality reduction.

Endothelial cells from the vehicle and ven/aza experimental conditions were integrated using the *Seurat* v. 5 integration strategy, resulting in an integrated PCA space (20 components) and its further embedding using Uniform Manifold Approximation and Projection (UMAP). The integrated PCA space was used as input for a Shared Nearest Neighbor (SNN) graph on which multi-resolution Leiden clustering was run, with resolution = 0.6 being chosen for concordance with reference-based labels. The new clusters were further annotated with a second run of *SingleR* reference-based annotation, using a murine bone marrow endothelial cell-specific dataset (*) resulting in the identification of one Arteriolar EC cluster, 4 Sinusoidal ECs, 2 Type R ECs, and 1 Type H EC cluster.

Cells from the recovery dataset were projected onto the vehicle and ven/aza ones using the *symphony*/*harmony* algorithms, so that recovery cells could be annotated by projection.

For differential expression (DE), which was only performed for ven/aza and vehicle data as they belong to the same experimental batch, clusters were pseudobulked within each condition and mouse, resulting in n = 3 pseudobulk replicates for vehicle clusters and n = 4 pseudobulk replicates for ven/aza clusters. Then, *pseudobulk DGE* from *scran* was run generating DE results between ven/aza and vehicle for each cluster separately. Genes ranked by log2(fold change) in each cluster were used for Gene Set Enrichment Analysis (GSEA) using Reactome pathways.

For differential abundance, *miloR* and *miloDE* were used on the *symphony*-integrated space generating fine grained condition-dependent shifts in cell type abundance. For differential cell-cell interaction in ven/aza and vehicle, the *multi-nichenet* R package was used using default parameters.

## Results

### Ven/aza treatment causes significant remodelling of the BM vasculature

**Fig. 1:**
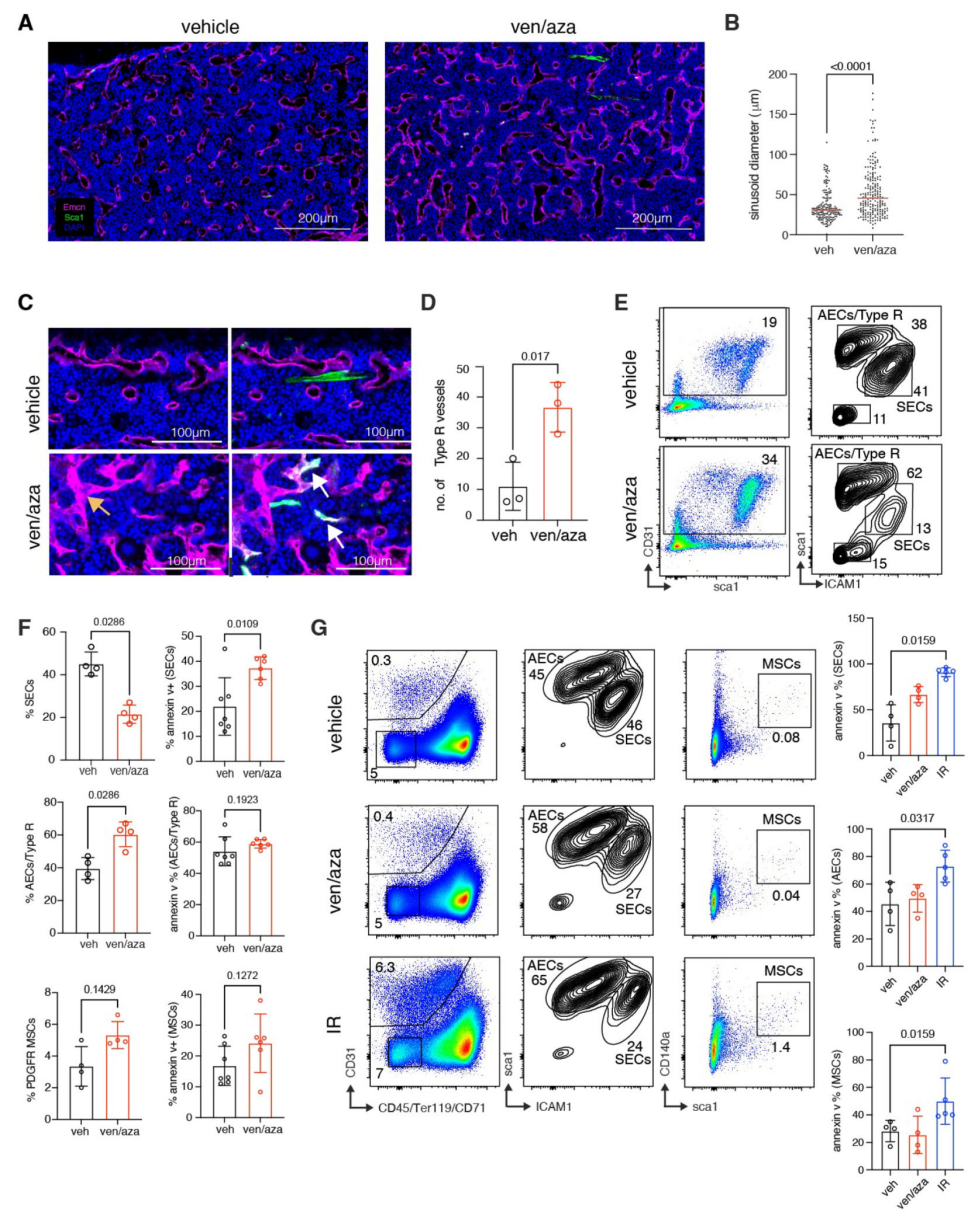
Ven/aza combinational therapy induces selective damage to the bone marrow vasculature. (**A**) Immunofluorescent imaging of vehicle control and ven/aza treated mice stained with Endomucin (Emcn), Sca1 and DAPI. (**B**) Quantification of sinusoid diameter from 3 slides from individual mice from each condition analyzed. (**C**) Immunofluorescent imaging displaying irregular sinusoids (orange arrow) and emergence of Type R vessels (white arrow) in ven/aza treated mice. (**D**) Quantification of Emcn^+^Sca1^+^ Type R vessels from immunofluorescent imaging. (**E**) Representative flow cytometry analysis of the EC compartment from vehicle and ven/aza treated mice. **(F)** Quantification of SEC and AEC or Type R (in %) in addition to percentage of apoptotic cells (annexin v^+^ %) in vehicle and ven/aza treated mice as assessed by flow cytometry analysis. Error bars indicate the S.D from three independent experiments, with a minimum of 3 slides analyzed. (**G**) Representative flow cytometry plots comparing the impact of ven/aza and irradiation on the EC and MSC compartments, in addition to quantification of the % of cells undergoing apoptosis (annexin v^+^ %) in each cell population. Error bars indicate the S.D from four individual mice in each condition. Mann Whitney test was performed to determine p value between comparisons.

To assess the impact of ven/aza on BME, immunodeficient mice were treated with the combinational therapy for two weeks and the BM cells were isolated for phenotypical characterization. Immunofluorescent imaging of femur sections revealed striking changes to the BM vasculature organization, including significant dilation of bone marrow sinusoids (Fig. 1A and B) and irregular morphology of sinusoidal endothelial cells (SECs) in ven/aza treated mice (Fig. 1C, orange arrow and 1D). Flow Cytometry analysis was also performed to further characterize the impact of ven/aza therapy on the BME. Consistent with our imaging data, ven/aza treatment had the greatest impact on the SEC population, resulting in a two-fold reduction in the percentage of SECs, coupled with a significant increase in SECs undergoing apoptosis (Fig 1E and 1F). Conversely, ven/aza therapy did not induce increased apoptosis in the AEC/Type R ECs or the PDGFR^+^ mesenchymal stroma cells (MSCs) in the BME (Fig 1F).

To assess whether the damage induced by ven/aza to the BME was similar to other therapeutic interventions, the BM of ven/aza treated mice was compared to those that underwent a single dose of sublethal irradiation. Interestingly, whereas ven/aza therapy only induced apoptosis and depletion of the SECs, irradiation resulted in increased apoptosis in both SEC/AEC as well as the MSC subpopulations (Fig 1G). Together, this data demonstrates that ven/aza therapy induces significant and selective remodelling of the BM vascular niche, resulting from selective depletion of SECs. To ascertain that the selective impact on SECs upon ven/aza treatment was not confined to immunodeficient mice, we also performed similar experiments using C57BL/6 mice and observed similar phenotypes as described (Suppl. Fig. 1A).

### Ven/aza therapy induced damage to the BME is restricted to the SEC compartment

**Fig. 2:**
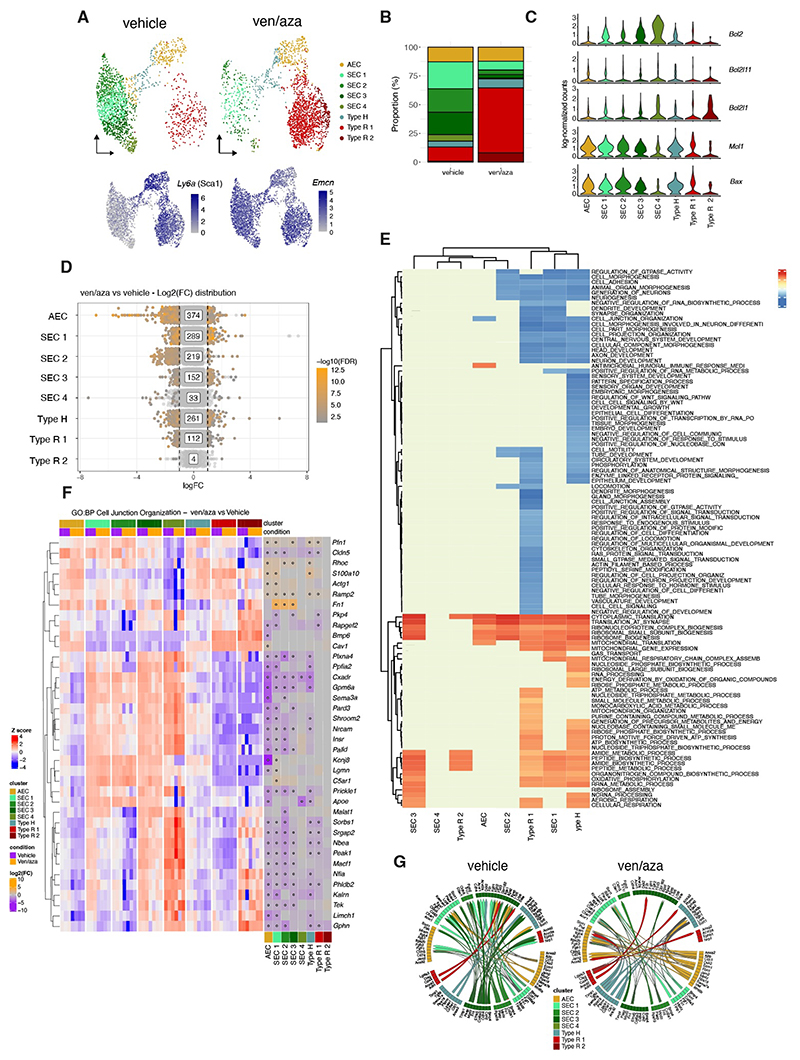
Structural damage of BM vasculature is coupled with transcriptional reprogramming of BM ECs and defects in HSPC homing. (**A**) UMAP visualization of color-coded BM niche EC clusters (n=8) in single-cell RNA-seq data, as well as expression levels of *Ly6a* (*Sca-1*) and *Emcn* expressed in Type R EC clusters. (**B**) Proportion plot displaying cell type composition in vehicle and ven/aza EC compartment. (**C**) Stacked violin plot of expression levels of apoptosis related genes (including *Bcl2, Bcl2l11, Bcl2l1, Mcl1* and *Bax*) in the EC clusters from vehicle control mice. (**D**) Stripchart displaying differential gene expression between conditions in each cluster. Points colored with a grey to orange gradient show statistical significance (according to FDR-adjusted p-values) where absolute log2(fold change) (log2(FC)) is above 1 and FDR-adjust p-value is below 0.05. (**E**) Normalized Enrichment Score (NES) matrix for Gene Set Enrichment Analysis (GSEA) per-cluster of DE results from ven/aza vs vehicle-treated conditions for the EC compartment. (**F**) Differentially Expressed (DE) Heatmap of genes between vehicle and ven/aza conditions along the cell junction (Gene Ontology gene set). Purple colored boxes signify downregulation in the ven/aza condition, whereas orange boxes signify upregulation. DE for a given gene that is statistically significant between conditions is marked with a dot in the centre of each box in the right-hand side heatmap. (**G**) Chord diagrams displaying the top 50 ranked ligand-receptor interactions in the EC compartment in vehicle and ven/aza treated mice.

To confirm our immunofluorescent imaging and flow cytometry-based characterization of the BM niche, we performed single-cell RNA sequencing (scRNAseq) on (CD45/Ter119/CD71)^neg^ sorted. BM niche cells to assess the transcriptional impact ven/aza has on the BME. Using 10x genomics, single cells first underwent rigorous quality control (QC) (Suppl Fig. 1B-F), and cells that passed QC were then annotated using a mouse BM stroma dataset as a reference (1), leading to the identification of several stromal populations (Suppl. Fig 1G). Given the high capture rate of endothelial cell (EC) populations, and the specific impact observed in the BM vasculature, we further annotated them using a recent, highly resolved mouse bone marrow EC dataset (12), which allowed us to reclassify ECs as arteriolar endothelial cell (AEC) Type H and Type R vessels, along with multiple sinusoidal endothelial cell (SEC) clusters (Suppl. Fig. 1G and I). Analysis of cell proportions in vehicle and ven/aza specific uMAPs indicated that cell type composition of endothelial populations differed significantly between both conditions (Fig. 2A and B). Due to the low capture of the different mesenchymal stromal cell (MSC) sub-populations in the initial scRNA-seq data set, we decided to perform a second scRNA-seq study by focusing the analysis on MSCs (Suppl. Fig. 2A and B). Analysis of their expression profile of niche-associated genes revealed that Adipo-CAR and Osteo-CAR expressed highest levels of *Cxcl12, Kitl* and *Lepr* expression (Suppl. Fig. 2C) and thus most likely represent the LepR^+^ MSC population (13). Although we observed that proportion of cells denoted as NG2^+^-MSCs and Osteo-CAR were slightly reduced in the ven/aza group compared to the control (Suppl. Fig. 2B), the proportion of LepR^+^ Adipo-CAR cells remained relatively unchanged (Suppl. Fig. 2B). Importantly, there was little DE genes in these MSC populations following ven/aza treatment (Suppl. Fig. 2D), in addition to no changes in the expression of important niche factors such as *Cxcl12* and *Kitl (*Suppl. Fig. 2E and F), suggesting that ven/aza has minimal impact on the MSC compartment.

Given that the most striking changes were observed in the EC compartment, we decided to focus on the BM vascular niche components in greater detail through differential abundance (DA) analysis (13) (Suppl. Fig. 1J and K), a robust statistical method utilized to assess the differences in abundance of cell populations between both conditions (details of analysis found in Suppl Materials and Methods). Most notably, DA revealed a significant underrepresentation of all SEC clusters in ven/aza treated mice, but no changes in AEC abundance (Suppl. Fig. 1J and K). Interestingly, analysis of the apoptosis-related gene expression profile revealed comparatively high expression of the venetoclax target *Bcl2* in the SEC clusters at homeostasis relative to other EC and stromal cell subtypes (Fig. 2C and Suppl Fig. 2F), thus providing a likely explanation as to why SECs may be particularly sensitive to ven/aza therapy. Unexpectedly, ven/aza treated mice also had a significantly higher proportion of Type R vessels, a recently reported post-arteriolar vessel-type typically localised in the trabecular and endosteal regions of the bone. As previously published, Type R vessels uniquely express *Dach1* (Suppl. Fig. 1I), endomucin (*Emcn*) and Sca-1 (*Ly6a*) (Fig. 2A). Using our immunofluorescent imaging, we confirmed the emergence of Emcn^+^Sca-1^+^ vessels primarily in the endosteal regions of femurs collected from ven/aza treated mice (Fig. 1C, white arrows and Fig. 1D), which likely correspond to the Type R vessels.

### Transcriptional reprogramming by ven/aza does not greatly impact expression of niche factor genes

To investigate whether cellular remodelling of the BM vasculature was coupled with transcriptional reprogramming of the EC compartment, cluster-level differential expression (DE) analysis with pseudo-bulking was performed between vehicle and ven/aza treated conditions. Notably, amongst all BME cell type captured, highest number of DE genes was found primarily in the vascular niche related clusters including the AEC cluster (374 DE genes), SEC clusters 1-3 (289, 219 and 152 DE genes respectively) as well as both Type H (261 DE genes) and Type R vessel (112 DE genes) clusters (Fig. 2D and Suppl. Table 1). Conversely, other stromal subtypes showed very little transcriptional reprogramming in response to ven/aza (Suppl. Fig. 1H and 2D), indicating an EC-specific impact of ven/aza on the BME. Gene Set Enrichment Analysis (GSEA) (Fig. 2E) revealed global changes to energy metabolism amongst all EC compartments in ven/aza compartments (SECs, AECs, Type H and Type R ECs), while SEC 1, Type H and Type R (12) show a down-regulation of Rho GTPase signalling, a key regulator of cell-cell junctions. AEC and SEC clusters saw the greatest number of DEG genes between conditions, whereas Type R EC clusters showed comparatively less therapy-induced transcriptional changes (Fig. 2D). Surprisingly, despite the significant transcriptional reprogramming of the vascular niche in response to ven/aza therapy, only modest changes were observed in niche factor genes typically associated with hematopoietic stem cell maintenance and survival (Suppl. Fig. 3B and C), such as *Cxcl12* and *Kitl* in all cell types that typically express both cytokines (Suppl. Fig 2E). There was a slight downregulation of Notch ligand family genes in the SEC clusters, including *Notch1, Dll4* as well as *Jag1* and *Jag2* in ven/aza treated mice, however many of these were not statistically significant (Suppl. Fig. 3C).

### Ven/aza induces breakdown in cell-cell communication and vascular leakiness in the BM vasculature

Although the expression of niche factor genes remains relatively unchanged in response to this therapy, gene set enrichment analysis of DE genes revealed a significant downregulation of cell junction and cell adhesion-related pathways across most of the EC clusters (Fig. 2E). Cell junction organization genes were particularly sensitive to ven/aza treatment (Fig. 2F), with widespread downregulation of genes across the SEC, AEC, Type H and to a lesser extent, Type R EC clusters. Cell adhesion was also compromised in response to therapy across all EC clusters (Suppl. Fig. 3A).

Given the significant downregulation of cell junction/cell adhesion related genes and Rho GTPase pathways across the EC compartments, we investigated through MultiNicheNet the differential cell-cell interactions (CCI) among murine ECs in response to ven/aza treatment (14). The algorithm prioritizes ligand-receptor (LR) interactions based on the differential expression of the genes coding for proteins involved in the physical interaction, and the targets of the ligand(s). Consistent with GSEA and DE analysis, the estimation of LR activity in receivers (clusters that express the receptors for ligands expressed by other clusters, and whose targets expression changes in response to changes in incoming ligands) showed a significant breakdown of CCI in the SEC compartments in response to ven/aza treatment (Fig. 2G and Suppl. Table 2). Interestingly, despite the significant reprogramming also observed in the AECs, there was instead a gain in highly differential outgoing CCI from the AEC cluster in response to therapy (16 in ven/aza treatment vs 8 in control amongst the top 50 differential interactions).

Among significantly changing interactions between ECs upon ven/aza treatment, the mpdz-claudin 5 (Cldn5) interaction in SECs is notable for its connection to vascular integrity. Indeed, Cldn5 has been previously reported as a critical junction protein for the maintenance of the blood-brain barrier (BBB), and its downregulation increases BBB permeability (15). Moreover, human MPDZ is also involved in angiogenesis and vascular integrity through its interaction with DLL4, a Notch ligand (16, 17). Another notable interaction between bone morphogenetic protein 4 (Bmp4), secreted by SECs, and activin receptor 2a (Acvr2a), expressed by Type R ECs, is reduced upon ven/aza treatment. Bmp4 is known to exert pro-angiogenic effects by destabilizing cell-cell junctions in ECs, while Acvr2a, a type II BMP receptor, balances TGF and BMP signalling (18).This interaction in vehicle-treated mice may favour the remodelling action of Type R ECs, which is greatly reduced upon ven/aza treatment.

**Fig. 3:**
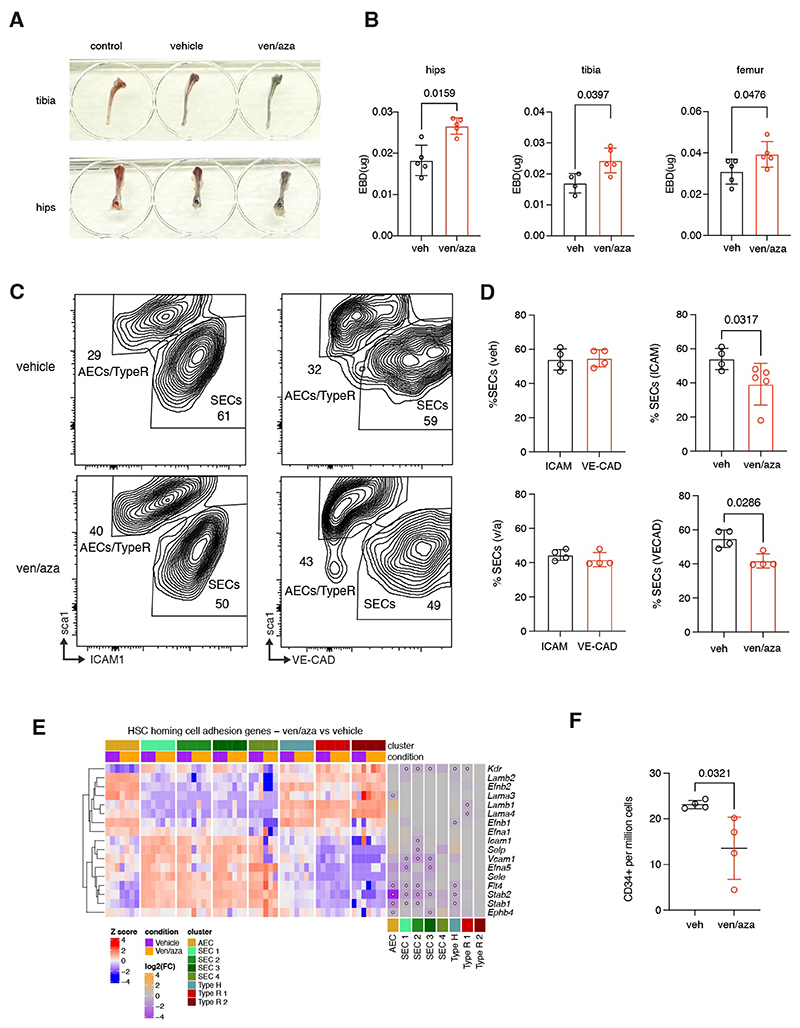
Ven/aza treatment causes major disruption to vascular integrity and EC adhesion in the BM niche (**A**) Representative images of tibia and hip bones collected from vehicle, ven/aza treated and control mouse not injected with EBD) mice injected with EBD 3 hours prior to culling. (**B**) Quantification of amount of EBD analyzed in the hips, tibia and femur bones of veh and ven/aza treated mice. Each point represents the average amount of EBD detected per pair of bones in each individual mouse. (**C**) Representative flow cytometry plots assessing the impact of ven/aza on VE-CAD expression via intravenous injection of VE-CAD antibody prior to cull. Population was pregated on mCD45^-^,Ter119^-^,CD71^-^,CD31^+^ cells. (**D**) Statistical comparison of the % of SECs in the BM as determined by ICAM-1 or VE-CAD expression in vehicle and ven/aza treated mice. (**E**) DE heatmap of adhesion genes related to HSC homing in ven/aza and vehicle-treated mice. (**F**) Quantification of CD45+CD34^+^ HSPCs that homed to the BM niche as assessed by flow cytometry. Mann Whitney test was performed to determine p value between all comparisons.

To assess whether the loss of cell-cell interaction and downregulation of adhesion/tight junction pathways in the SECs resulted in increased vascular permeability in the BM, we performed evans blue dye (EBD) extravasation assay on ven/aza treated mice. In comparison to the vehicle treated counterpart, two weeks of ven/aza therapy resulted in increased EBD leakage into the BM of femurs, tibiae and iliac crests (Fig. 3A and B), signifying treatment-induced disruption of vascular integrity. V-Cadherin (VE-CAD) is a key component of EC junction formation and is typically highly expressed in SECs (19).In addition to the EBD assay, we also intravenously injected ven/aza treated mice with VE-CAD prior to culling to accurately assess whether the combinational therapy also reduced the expression of the junction protein essential for vascular integrity. Interestingly, two weeks of ven/aza therapy also resulted in a significant reduction in SECs as identified by VE-CAD expression (Fig 3C and D). Together, these functional results coupled with the transcriptional changes support the breakdown phenotype in EC networks with a significant remodelling and thus, compromising the BM vasculature in response to ven/aza therapy.

### Ven/aza induced damage and reprogramming of SECs results in defects in human HSPCs homing

SECs are heavily associated with leukocyte trafficking and homing of transplanted HSPCs to the BM niche (20, 21). Interestingly, in addition to the ven/aza induced reduction of SECs, there was also a significant downregulation of genes associated with HSC homing to the vasculature in the remaining SECs, including *Vcam1* (SEC 1-3); *Icam1* in SEC 2; *Stab1* in SEC 1-2, *Stab2* in SEC 1-3 and *Selp* in SEC 2 (Fig. 3E). Flow cytometry analysis also confirmed reduced expression of these adhesion molecules (Suppl. Fig. 3D). *Vegfr2 (Kdr)* expression in SECs is also important for HSC engraftment, such that treatment with mAb to Vegfr2 post-irradiation causes a reduction in HSC reconstitution (22). Interestingly, ven/aza treatment also induced a significant downregulation of both *Vegfr2 (Kdr)* and *Vegfr3 (Flt4)* in multiple SEC clusters (Fig. 3E), suggesting that the role of SECs in HSPC homing may be negatively impacted. Given the downregulation of several adhesion genes essential for facilitating HSPC homing coupled with the disruption of vascular integrity in the BM, we performed an *in vivo* homing assay to assess whether ven/aza induced destruction of vascular integrity resulted in defects in donor-derived HSPC homing. Following 48h hours post ven/aza treatment, mice injected with human CD34^+^ HSPCs had almost a ~2-fold lower (mean of HSPCs in veh = 23.13, ven/aza = 13.56) frequency of HSPCs home to the BM compared to vehicle control mice (Fig. 3F), thus supporting our hypothesis that ven/aza induced damage to the SECs significantly compromises the effective homing of donor-derived HSPCs to the BM niche.

### Remodelling of the BM vasculature is ameliorated three weeks post therapy

**Fig. 4:**
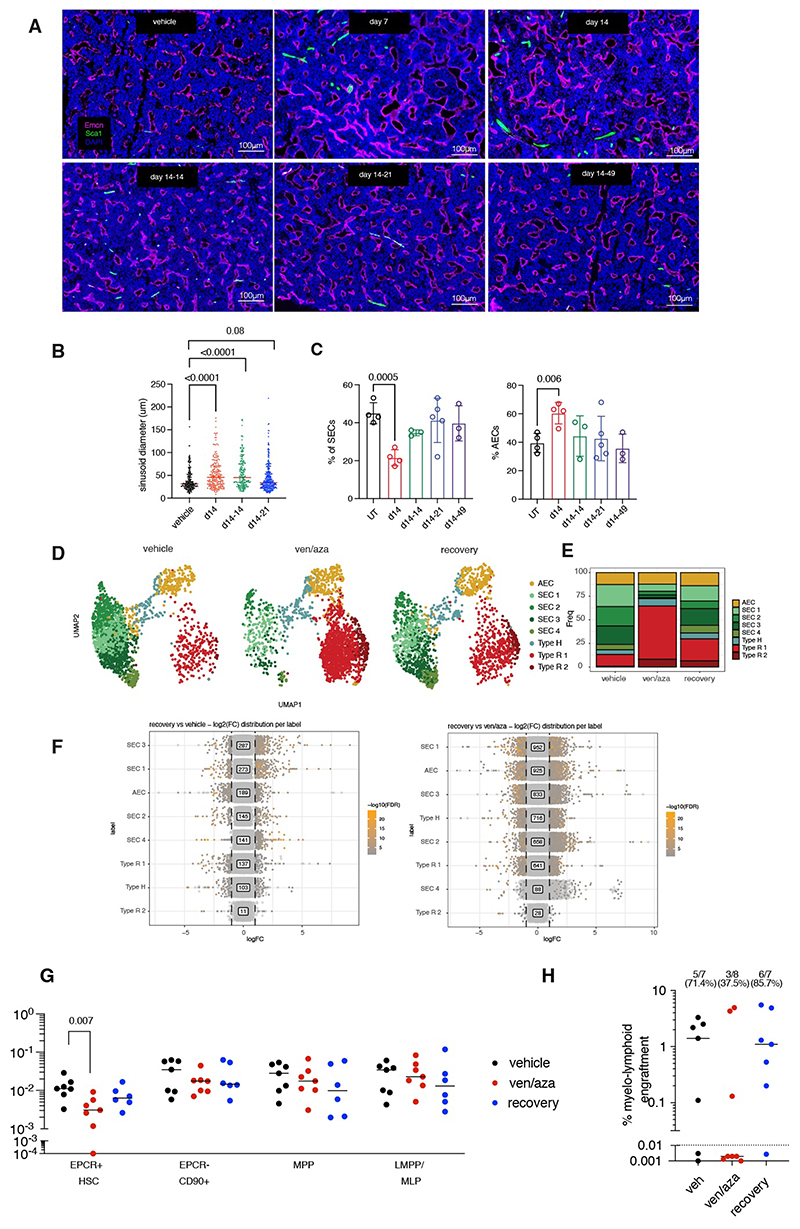
BM vascular niche undergoes partial recovery following several weeks post ven/aza treatment (**A**) Representative images of femur sections immunostained with Emcn, Sca-1 and DAPI. (**B**) Quantification of sinusoidal diameter across all timepoints. (**C**) Flow cytometry analysis of % of SEC and % of AEC within CD45^-^CD71^-^Ter119^-^CD31^+^ EC population across all the indicated timepoints. Datapoints were collected over three independent experiments and minimum of three slides analyzed per timepoint. (**D**) UMAP visualization of bone marrow EC clusters across the veh, ven/aza and recovery mice after data integration. (**E**) Proportion plot demonstrating cell type composition across all the indicated timepoints. (**F**) Stripchart of DE genes between recovery vs vehicle and recovery vs ven/aza conditions for each of the EC clusters. (**G**) Quantification of EPCR^+^ (CD201^+^) HSCs, MPPs and LMPP/MLPs across all conditions as assessed by flow cytometry. Each dot represents a mouse and median bars are shown. (**H**) percentage of human engraftment in secondary recipient mice transplanted with human CD34^+^ collected from vehicle, ven/aza or recovery mice. Each dot represents a mouse and median bars are shown. Mann Whitney test was performed to determine p value between all comparisons.

Based on the striking ven/aza induced damage to the vasculature, we sought to investigate whether this short-term ven/aza treatment inflicted longer term damage to the BM niche. For that, we treated mice with ven/aza for two weeks, followed by periodical assessment of the BM vasculature. Re-organisation of sinusoids (Fig. 4A and B) and re-emergence of ICAM-1^+^ SECs via flow-cytometry analysis was only observed three weeks post-treatment (Fig. 4C and Suppl. Fig. 4A). Intriguingly, scRNAseq of the BME of mice 50 days post-treatment revealed only partial recovery of BM EC cell type composition and SEC abundance upon DA analysis (Fig. 4D and E, Suppl. Fig. 4B-E). Furthermore, in contrast to the near absence in vehicle control mice, Type-R EC 2 neighborhoods that expanded in response to ven/aza treatment remained present in the BM vasculature (Suppl. Fig. 4B-4E). The transcriptional profile of ECs from recovery mice resembled vehicle ECs more than ven/aza ECs, however there remained considerable transcriptional differences between ECs from these conditions (Fig. 4F), including notable differences in metabolism related pathways (Suppl. Fig. 4F). Together, this data suggests that ven/aza damage was partially reversible, with significant yet incomplete recovery of the BM vasculature following several weeks post treatment.

### Engraftment of highly primitive HSCs is partially rescued with the restoration of the SEC compartment

To assess whether the partial recovery of the SEC compartment was reflected functionally, we transplanted CD34^+^ HSPCs into vehicle, ven/aza treated mice, and mice left to recover three weeks post treatment. Surprisingly, no difference in total human engraftment and lineage outputs was observed between all-experimental conditions 9 weeks post-transplantation (Suppl. Fig. 5A-B). However, further analysis of the donor-derived HSPCs revealed a 4-fold decrease (p value = 0.007) in the percentage of EPCR^+^ HSCs in mice transplanted with HSPCs shortly after ven/aza treatment (Fig. 4G). As a result of this, human HSPCs isolated from ven/aza treated mice were less effective in engrafting in secondary recipient mice (5 out of 7 mice from vehicle control, 3 out of 8 from ven/aza treated) (Fig. 4H), further demonstrating that the disruption of vascular integrity induced by ven/aza therapy compromises the long-term engraftment of EPCR^+^ HSCs.

Interestingly, the frequency of EPCR^+^ HSCs engrafted in mice transplanted with HSPCs three weeks post treatment (‘recovery’ mice) remained 1.7-fold less than the control, indicating that the incomplete transcriptional recovery of the endothelium was reflected in its functional output (Fig. 4G). However despite this modest defect in EPCR^+^ HSCs, human cells derived from recovery mice was able to successfully engraft in secondary recipient mice at similar success rates (6 out of 7 mice) observed in vehicle control mice (5 out of 7 mice) (Fig. 4H), indicating that the recovery of the SEC compartment is critical for the effective engraftment of a subset of highly regenerative HSCs with multilineage differentiation capacity (23, 24).

## Discussion

Venetoclax-based therapies are increasingly used clinically to treat hematological malignancies such as AML and multiple myeloma, in part due to their low cytotoxicity in comparison to conventional chemotherapies (7). Using humanised mice, we were able to characterize for the first time, the damage ven/aza causes to the BM vasculature primarily through the selective depletion of the SEC compartment. SEC sensitivity to therapy is not unique to ven/aza treatment, as evidence of SEC depletion has been showcased in other therapeutic interventions such as chemotherapy, irradiation and myeloablation (16). Notably, such damage to the SEC population is likely a result of treatment-induced inflammation of the BM niche (16, 25). Furthermore, damage caused to the BME is not specific to SECs, but instead negatively impacts all EC and stromal cell compartments. Conversely, ven/aza treatment seemingly does not trigger overt inflammation in the BME. Rather, ven/aza therapy causes selective damage primarily to the SEC compartment, presumably due to their comparatively high levels of Bcl2 expression. Hence an important distinction between irradiation or chemotherapy compared to ven/aza treatment is that the latter does not greatly impact other ECs or stromal cell populations.

ECs plays a critical role in regulating hematopoiesis, however distinguishing between the cell-specific roles of each EC subtype remains controversial, in part due to the growing understanding of the true heterogeneity in both HSCs and the EC compartment. Cxcl12 and Kitl secreting MSCs are also thought to play a critical role in HSPC homing (26, 27). We found that ven/aza caused minimal damage to the MSC compartment and also very little change in Cxcl12 and Kitl expression in all the BM cell types that typically express both cytokines. Instead, we found that not only was ven/aza damage specific to SECs, but this alone was sufficient to negatively impact HSPC homing and engraftment of highly primitive HSCs. Hence surprisingly, it appears that damage to the vascular integrity rather than the expression of chemoattractants such as Cxcl12 and Kitl has a greater impact on HSC homing and engraftment of highly primitive HSCs. Therefore, these findings highlight the importance of SECs in HSPC homing and engraftment of LT-HSCs following ven/aza therapy and that failed or inefficient HSCT following other therapeutic interventions, may also be attributed primarily to the damage to the SEC compartment rather than the non-specific damage to the BME.

Ven/aza treatment also saw an unexpected expansion of a newly characterized vessel known as Type-R capillaries, accompanied by an increase in outgoing signalling from these cells. Although currently our understanding of Type-R capillaries is heavily associated with bone remodelling, the relationship between Type R vessels and HSPCs remains largely unknown. Therefore, whilst our data shows the breakdown of known interactions along the HSPC-SEC axis, we cannot exclude the possibility that the emergence of Type-R vessels could also contribute to impaired HSC engraftment and this warrants further investigation.

In conclusion, we have comprehensively characterized the ven/aza induced remodelling of the BM vascular niche along with its downstream impact on HSPC homing and engraftment of highly primitive EPCR^+^ HSCs. Hence, interventions that could maintain SEC integrity may become a key determinant in improving HSC reconstitution and patient outcomes for those that undergo BMT post therapy.

### Materials availability

All biological materials used in this study are available from the lead contact upon request or from commercial sources. This study did not generate new unique reagents.

### Lead contact

Further information and requests for resources and reagents should be directed to and will be fulfilled by the lead contact, Dominique Bonnet (Dominique.bonnet@crick.ac.uk)

## Supplementary Material

Suppl. Fig 1

Suppl Table 1

Suppl Table 2

## Data Availability

Single cell RNA-sequencing have been deposited at GEO and are publicly available as of the date of publication. Accession numbers is GSE297754.

## Key points

- The Venetoclax and azacitidine (ven/aza) combinational therapy causes significant cellular and transcriptional remodelling of the bone marrow vasculature
- Ven/aza induced damage to the bone marrow vasculature leads to impairments in donor-derived HSPC homing and engraftment of EPCR^+^ HSCs
