# Supplementary material for: Remodelling of the bone marrow vasculature induced by venetoclax and azacitidine damage
Source: Blood. Author manuscript; Available in PMC 2026 Feb 18. (PMC7618727; doi:10.1182/blood.2025030055)
Supplement: Suppl. Fig 1 [file EMS212453-supplement-Suppl__Fig_1.pdf]

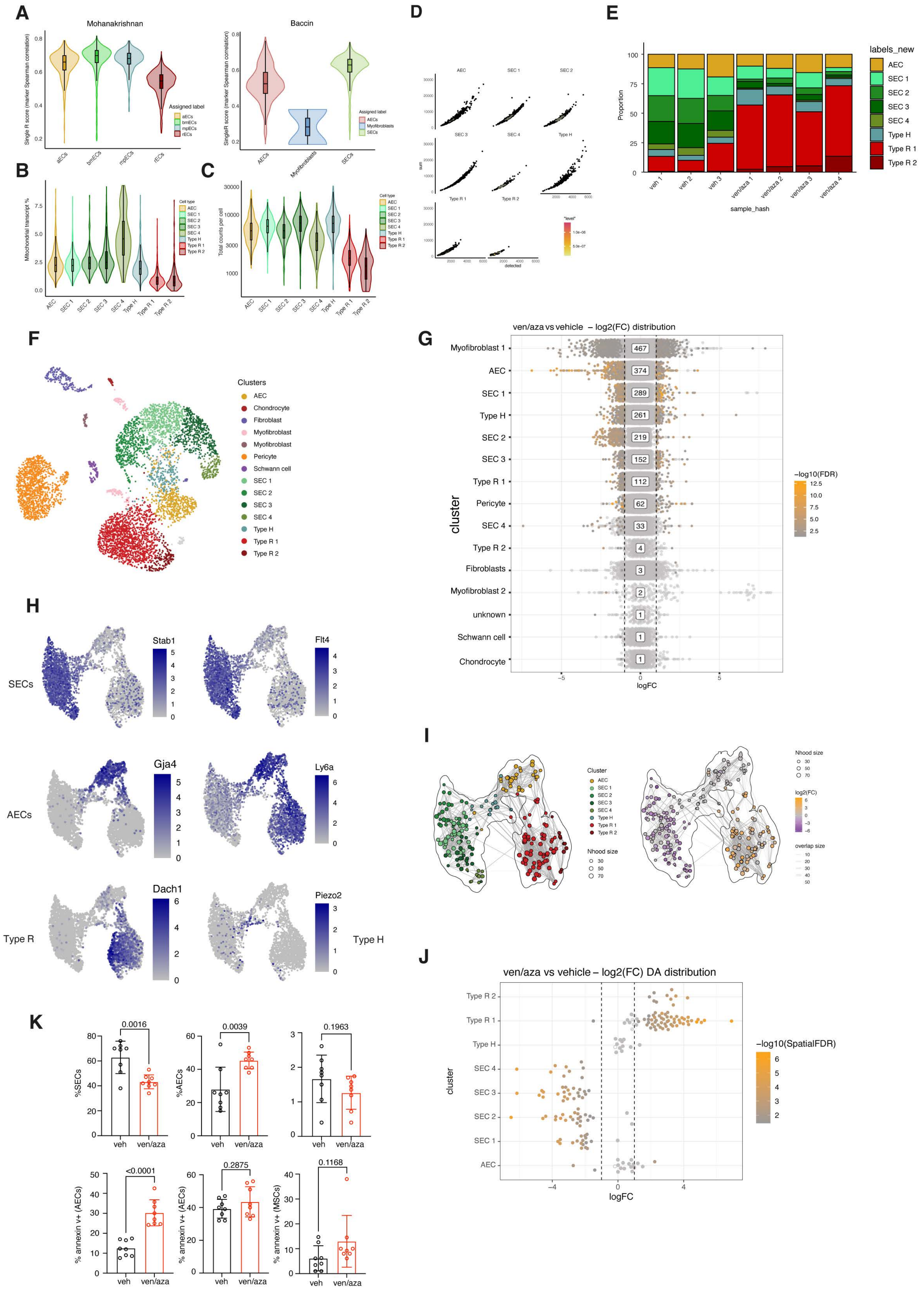
A
Mohanakrishnan
Single R score (marker Spearman correlation)
Assigned label
aECs
bmECs
mpECs
rECs
Baccin
SingleR score (marker Spearman correlation)
AECs
Myofibroblasts
SECs
B
Mitochondrial transcript %
Cell type
AEC
SEC 1
SEC 2
SEC 3
SEC 4
Type H
Type R 1
Type R 2
C
Total counts per cell
D
sum
detected
"level"
E
Proportion
sample_hash
veh 1
veh 2
veh 3
ven/aza 1
ven/aza 2
ven/aza 3
ven/aza 4
labels_new
F
Clusters
AEC
Chondrocyte
Fibroblast
Myofibroblast
Myofibroblast
Pericyte
Schwann cell
SEC 1
SEC 2
SEC 3
SEC 4
Type H
Type R 1
Type R 2
G
ven/aza vs vehicle – log2(FC) distribution
Myofibroblast 1
AEC
SEC 1
Type H
SEC 2
SEC 3
Type R 1
Pericyte
SEC 4
Type R 2
Fibroblasts
Myofibroblast 2
unknown
Schwann cell
Chondrocyte
467
374
289
261
219
152
112
62
33
4
3
2
1
1
1
cluster
logFC
−log10(FDR)
H
SECs
Stab1
Flt4
AECs
Gja4
Ly6a
Type R
Dach1
Piezo2
Type H
I
Cluster
Nhood size
log2(FC)
overlap size
J
ven/aza vs vehicle – log2(FC) DA distribution
cluster
logFC
−log10(SpatialFDR)
K
%SECs
0.0016
%AECs
0.0039
0.1963
% annexin v+ (AECs)
<0.0001
0.2875
% annexin v+ (MSCs)
0.1168
veh
ven/aza

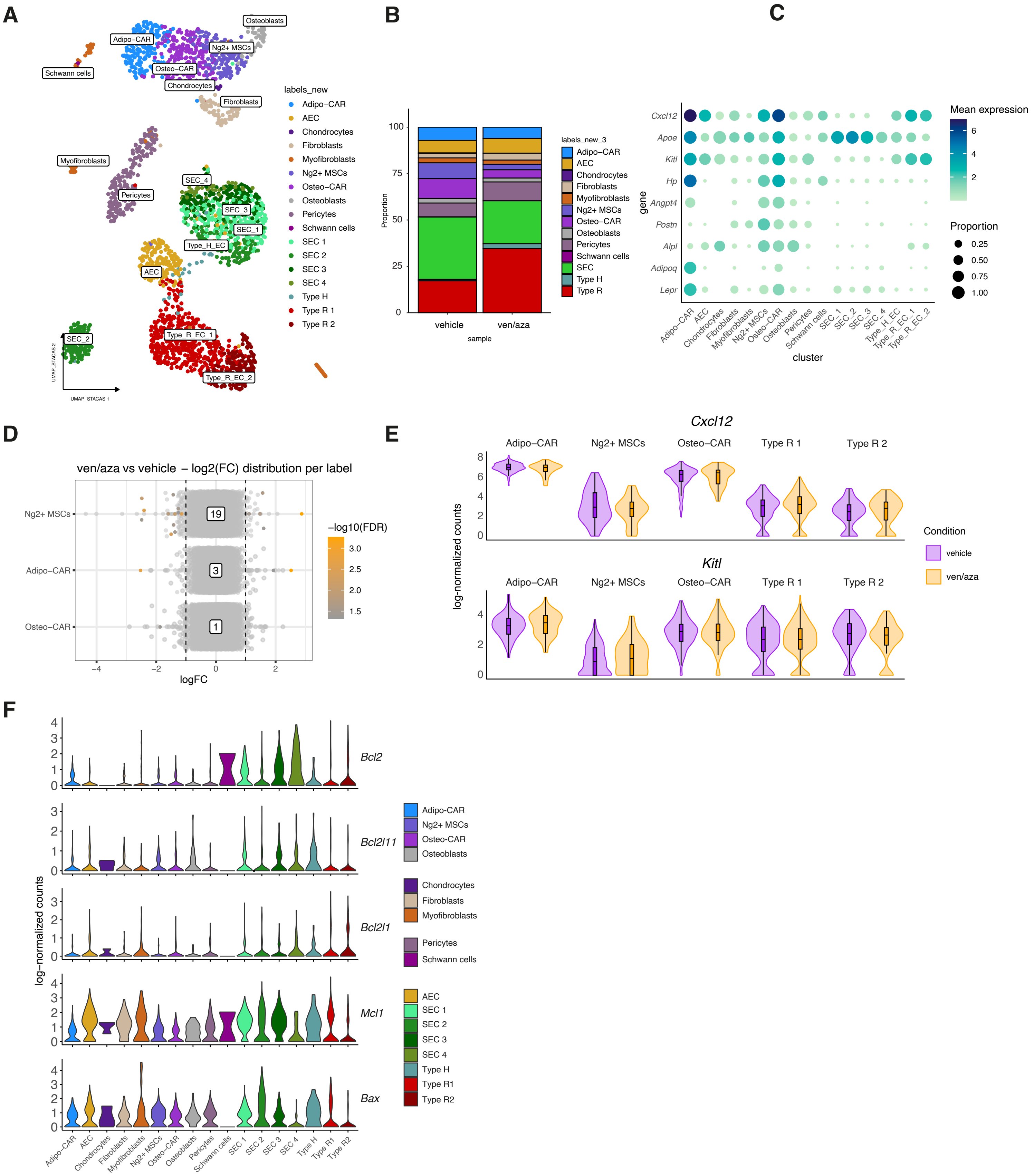
A
Osteoblasts
Adipo–CAR
Ng2+ MSCs
Schwann cells
Osteo–CAR
Chondrocytes
Fibroblasts
Myofibroblasts
Pericytes
SEC_4
SEC_3
SEC_1
Type_H_EC
AEC
SEC_2
Type_R_EC_1
Type_R_EC_2
UMAP_STACAS 2
UMAP_STACAS 1
labels_new
Adipo–CAR
AEC
Chondrocytes
Fibroblasts
Myofibroblasts
Ng2+ MSCs
Osteo–CAR
Osteoblasts
Pericytes
Schwann cells
SEC 1
SEC 2
SEC 3
SEC 4
Type H
Type R 1
Type R 2
B
Proportion
100
75
50
25
0
vehicle
ven/aza
sample
labels_new_3
Adipo–CAR
AEC
Chondrocytes
Fibroblasts
Myofibroblasts
Ng2+ MSCs
Osteo–CAR
Osteoblasts
Pericytes
Schwann cells
SEC
Type H
Type R
C
gene
Cxcl12
Apoe
Kitl
Hp
Angpt4
Postn
Alpl
Adipoq
Lepr
Adipo–CAR
AEC
Chondrocytes
Fibroblasts
Myofibroblasts
Ng2+ MSCs
Osteo–CAR
Osteoblasts
Pericytes
Schwann cells
SEC_1
SEC_2
SEC_3
SEC_4
Type_H_EC
Type_R_EC_1
Type_R_EC_2
cluster
Mean expression
6
4
2
Proportion
0.25
0.50
0.75
1.00
D
ven/aza vs vehicle – log2(FC) distribution per label
Ng2+ MSCs
Adipo–CAR
Osteo–CAR
19
3
1
–4
–2
0
2
logFC
–log10(FDR)
3.0
2.5
2.0
1.5
E
Cxcl12
Adipo–CAR
Ng2+ MSCs
Osteo–CAR
Type R 1
Type R 2
8
6
4
2
0
Kitl
Adipo–CAR
Ng2+ MSCs
Osteo–CAR
Type R 1
Type R 2
4
2
0
log–normalized counts
Condition
vehicle
ven/aza
F
Bcl2
Bcl2l11
Bcl2l1
Mcl1
Bax
log–normalized counts
Adipo–CAR
AEC
Chondrocytes
Fibroblasts
Myofibroblasts
Ng2+ MSCs
Osteo–CAR
Osteoblasts
Pericytes
Schwann cells
SEC 1
SEC 2
SEC 3
SEC 4
Type H
Type R1
Type R2
Adipo-CAR
Ng2+ MSCs
Osteo-CAR
Osteoblasts
Chondrocytes
Fibroblasts
Myofibroblasts
Pericytes
Schwann cells
AEC
SEC 1
SEC 2
SEC 3
SEC 4
Type H
Type R1
Type R2

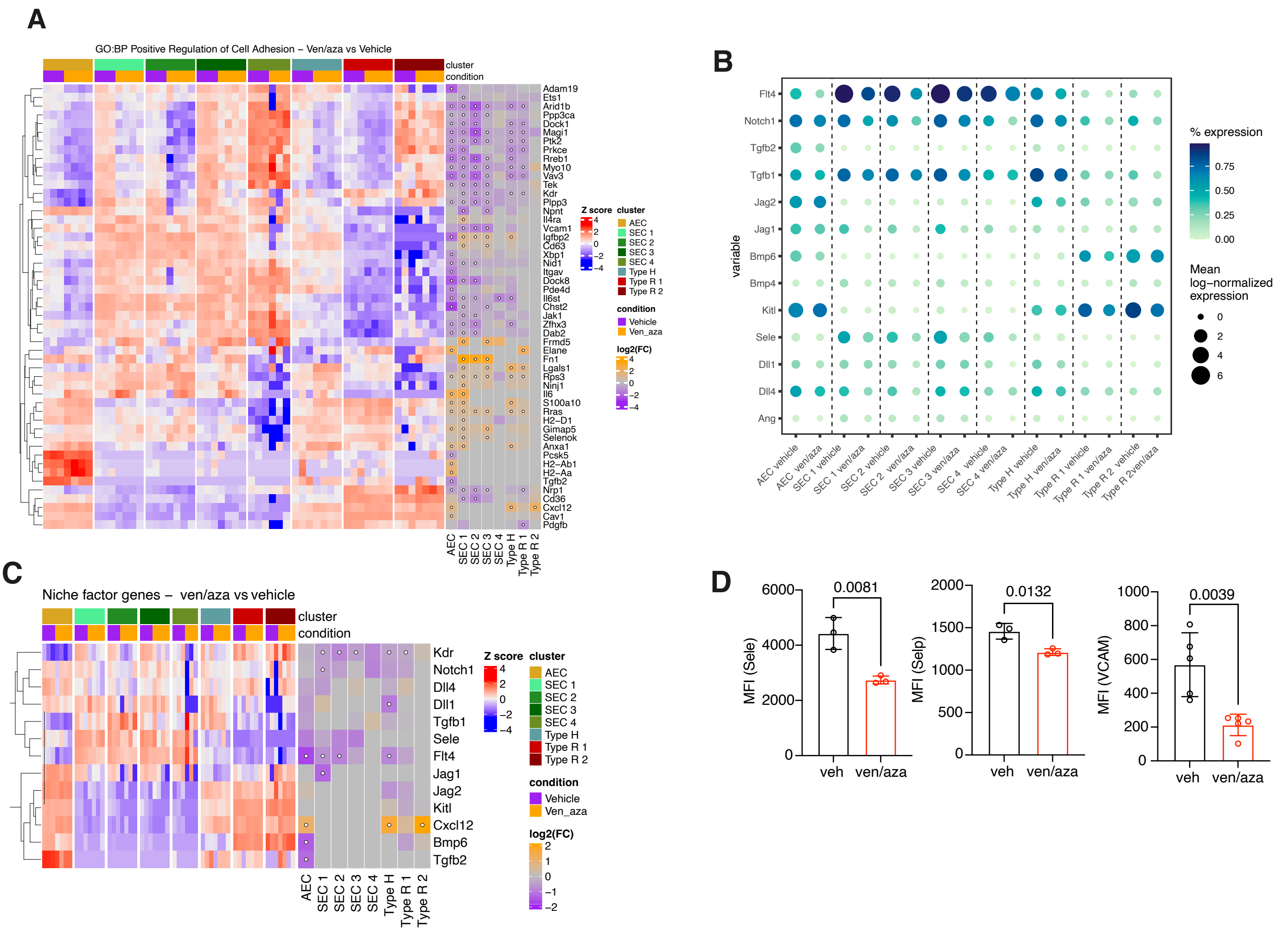
A
GO:BP Positive Regulation of Cell Adhesion – Ven/aza vs Vehicle
cluster
condition
Adam19
Ets1
Arid1b
Ppp3ca
Dock1
Magi1
Ptk2
Prkce
Rreb1
Myo10
Vav3
Tek
Kdr
Plpp3
Npnt
Il4ra
Vcam1
Igfbp2
Cd63
Xbp1
Nid1
Itgav
Dock8
Pde4d
Il6st
Chst2
Jak1
Zfhx3
Dab2
Frmd5
Elane
Fn1
Lgals1
Rps3
Ninj1
Il6
S100a10
Rras
H2–D1
Gimap5
Selenok
Anxa1
Pcsk5
H2–Ab1
H2–Aa
Tgfb2
Nrp1
Cd36
Cxcl12
Cav1
Pdgfb
AEC
SEC 1
SEC 2
SEC 3
SEC 4
Type H
Type R 1
Type R 2
Z score
4
2
0
−2
−4
cluster
AEC
SEC 1
SEC 2
SEC 3
SEC 4
Type H
Type R 1
Type R 2
condition
Vehicle
Ven_aza
log2(FC)
4
2
0
−2
−4
B
Flt4
Notch1
Tgfb2
Tgfb1
Jag2
Jag1
Bmp6
Bmp4
Kitl
Sele
Dll1
Dll4
Ang
variable
AEC vehicle
AEC ven/aza
SEC 1 vehicle
SEC 1 ven/aza
SEC 2 vehicle
SEC 2 ven/aza
SEC 3 vehicle
SEC 3 ven/aza
SEC 4 vehicle
SEC 4 ven/aza
Type H vehicle
Type H ven/aza
Type R 1 vehicle
Type R 1 ven/aza
Type R 2 vehicle
Type R 2ven/aza
% expression
0.75
0.50
0.25
0.00
Mean
log–normalized
expression
0
2
4
6
C
Niche factor genes – ven/aza vs vehicle
cluster
condition
Kdr
Notch1
Dll4
Dll1
Tgfb1
Sele
Flt4
Jag1
Jag2
Kitl
Cxcl12
Bmp6
Tgfb2
AEC
SEC 1
SEC 2
SEC 3
SEC 4
Type H
Type R 1
Type R 2
Z score
4
2
0
−2
−4
cluster
AEC
SEC 1
SEC 2
SEC 3
SEC 4
Type H
Type R 1
Type R 2
condition
Vehicle
Ven_aza
log2(FC)
2
1
0
−1
−2
D
MFI (Sele)
6000
4000
2000
0
0.0081
veh
ven/aza
MFI (Selp)
2000
1500
1000
500
0
0.0132
veh
ven/aza
MFI (VCAM)
1000
800
600
400
200
0
0.0039
veh
ven/aza

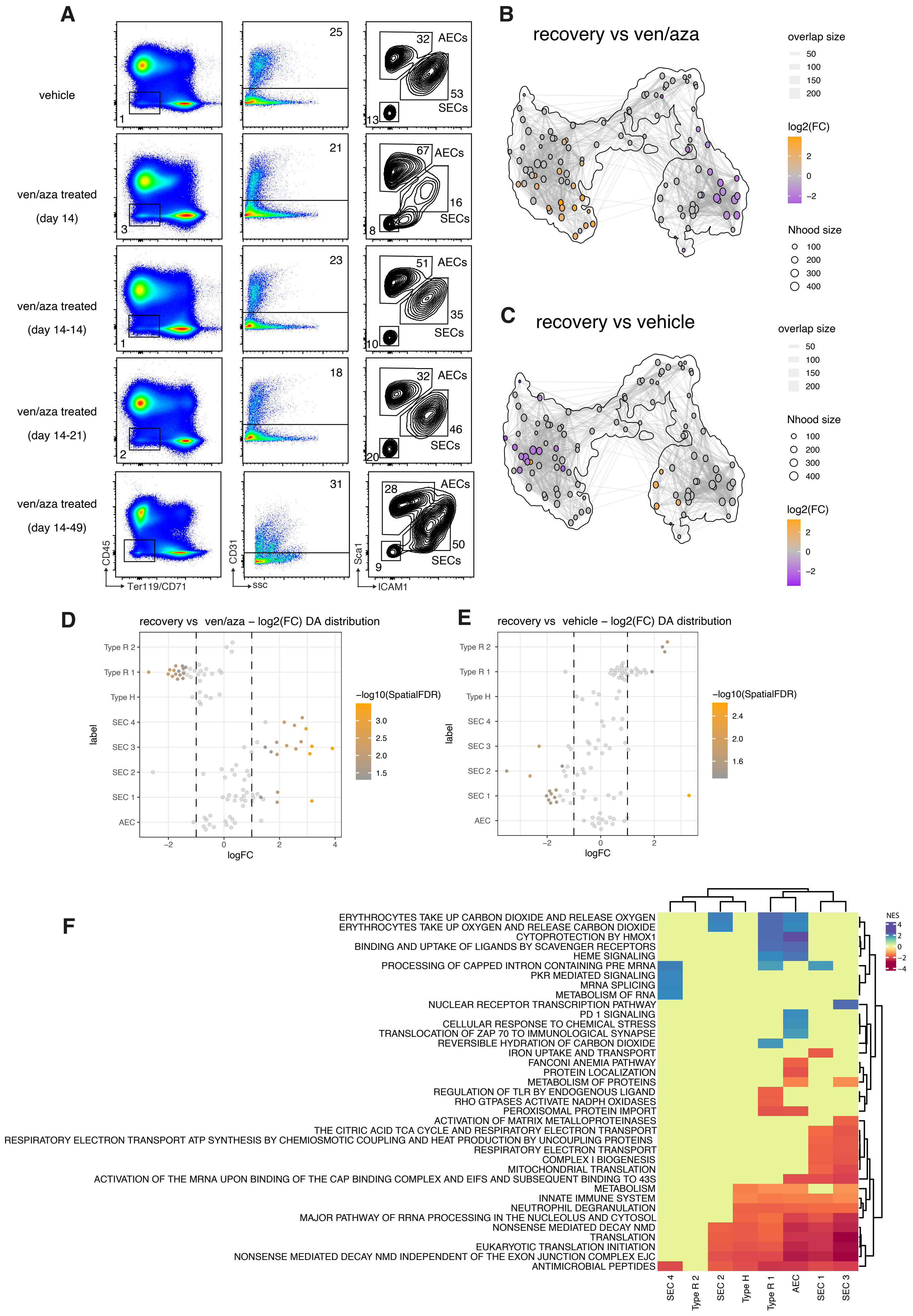
A
vehicle
ven/aza treated
(day 14)
ven/aza treated
(day 14-14)
ven/aza treated
(day 14-21)
ven/aza treated
(day 14-49)
CD45
Ter119/CD71
CD31
SSC
Sca1
ICAM1
AECs
SECs
1
25
32
53
13
3
21
67
16
8
1
23
51
35
10
2
18
32
46
20
31
28
50
9
B
recovery vs ven/aza
overlap size
50
100
150
200
log2(FC)
2
0
−2
Nhood size
100
200
300
400
C
recovery vs vehicle
overlap size
50
100
150
200
Nhood size
100
200
300
400
log2(FC)
2
0
−2
D
recovery vs ven/aza – log2(FC) DA distribution
Type R 2
Type R 1
Type H
SEC 4
SEC 3
SEC 2
SEC 1
AEC
label
logFC
−2
0
2
4
−log10(SpatialFDR)
3.0
2.5
2.0
1.5
E
recovery vs vehicle – log2(FC) DA distribution
Type R 2
Type R 1
Type H
SEC 4
SEC 3
SEC 2
SEC 1
AEC
label
logFC
−2
0
2
−log10(SpatialFDR)
2.4
2.0
1.6
F
ERYTHROCYTES TAKE UP CARBON DIOXIDE AND RELEASE OXYGEN
ERYTHROCYTES TAKE UP OXYGEN AND RELEASE CARBON DIOXIDE
CYTOPROTECTION BY HMOX1
BINDING AND UPTAKE OF LIGANDS BY SCAVENGER RECEPTORS
HEME SIGNALING
PROCESSING OF CAPPED INTRON CONTAINING PRE MRNA
PKR MEDIATED SIGNALING
MRNA SPLICING
METABOLISM OF RNA
NUCLEAR RECEPTOR TRANSCRIPTION PATHWAY
PD 1 SIGNALING
CELLULAR RESPONSE TO CHEMICAL STRESS
TRANSLOCATION OF ZAP 70 TO IMMUNOLOGICAL SYNAPSE
REVERSIBLE HYDRATION OF CARBON DIOXIDE
IRON UPTAKE AND TRANSPORT
FANCONI ANEMIA PATHWAY
PROTEIN LOCALIZATION
METABOLISM OF PROTEINS
REGULATION OF TLR BY ENDOGENOUS LIGAND
RHO GTPASES ACTIVATE NADPH OXIDASES
PEROXISOMAL PROTEIN IMPORT
ACTIVATION OF MATRIX METALLOPROTEINASES
THE CITRIC ACID TCA CYCLE AND RESPIRATORY ELECTRON TRANSPORT
RESPIRATORY ELECTRON TRANSPORT ATP SYNTHESIS BY CHEMIOSMOTIC COUPLING AND HEAT PRODUCTION BY UNCOUPLING PROTEINS
RESPIRATORY ELECTRON TRANSPORT
COMPLEX I BIOGENESIS
MITOCHONDRIAL TRANSLATION
ACTIVATION OF THE MRNA UPON BINDING OF THE CAP BINDING COMPLEX AND EIFS AND SUBSEQUENT BINDING TO 43S
METABOLISM
INNATE IMMUNE SYSTEM
NEUTROPHIL DEGRANULATION
MAJOR PATHWAY OF RRNA PROCESSING IN THE NUCLEOLUS AND CYTOSOL
NONSENSE MEDIATED DECAY NMD
TRANSLATION
EUKARYOTIC TRANSLATION INITIATION
NONSENSE MEDIATED DECAY NMD INDEPENDENT OF THE EXON JUNCTION COMPLEX EJC
ANTIMICROBIAL PEPTIDES
SEC 4
Type R 2
SEC 2
Type H
Type R 1
AEC
SEC 1
SEC 3
NES
4
2
0
−2
−4

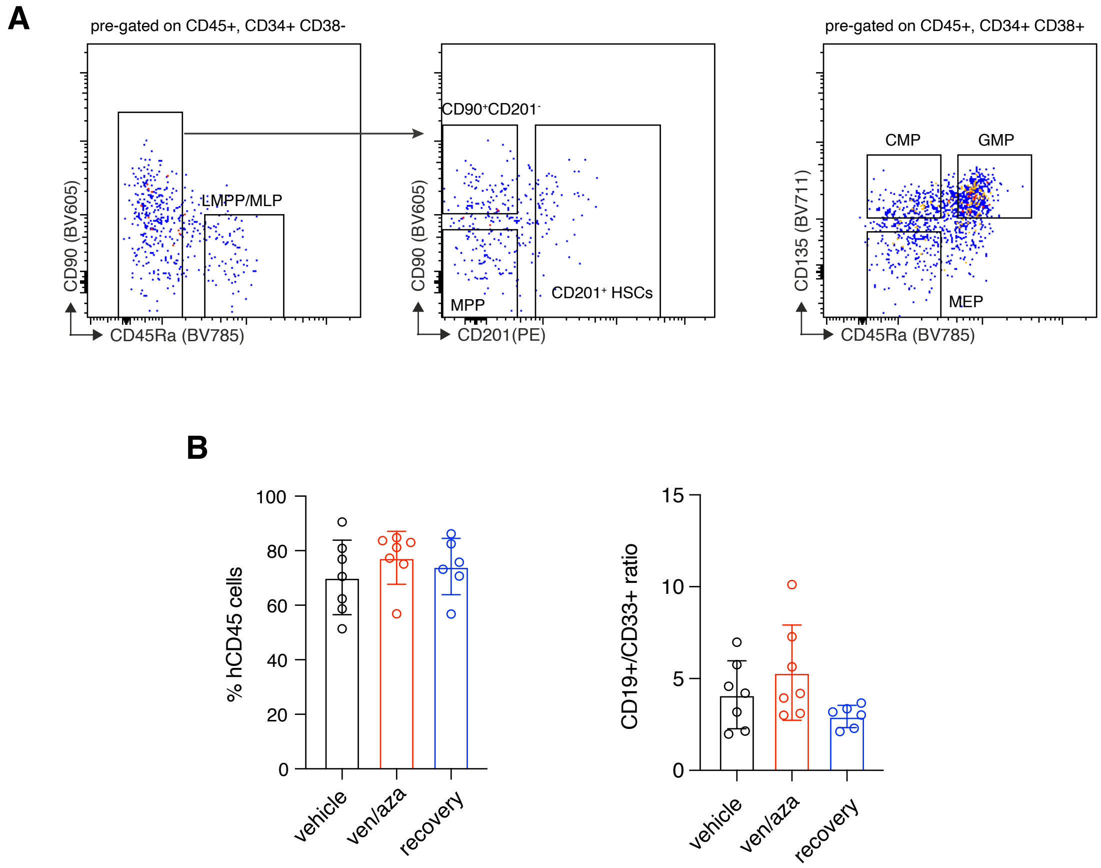
A
pre-gated on CD45+, CD34+ CD38-
CD90 (BV605)
CD45Ra (BV785)
LMPP/MLP
CD90+CD201-
CD90 (BV605)
CD201(PE)
MPP
CD201+ HSCs
pre-gated on CD45+, CD34+ CD38+
CD135 (BV711)
CD45Ra (BV785)
CMP
GMP
MEP
B
% hCD45 cells
100
80
60
40
20
0
vehicle
ven/aza
recovery
CD19+/CD33+ ratio
15
10
5
0
vehicle
ven/aza
recovery

Suppl. Fig. 1. **(A)** Quantification of SEC, AEC/Type R and PDGFR+ MSCs (in %) in addition to percentage of apoptotic cells (based off annexin v+ %) of each compartment inimmunocompetent C57/B6 mice. Error bars indicate the S.D from 8 individual mice in each condition. Mann Whitney test was performed to determine p value between comparisons. **(B)** Violin plots representing Confidence in reference-based cell type mapping (SingleR scores) using both the Baccin et al. (1) and the Mohanakrishnan et al. (12) datasets. **(C)** Percentage of mitochondrial transcript per cell type. **(D)**Total UMI counts per cell and total genes detected as violin plots, **(E)** and as scatterplots. **(F)** Barplot representing proportion of cell type per condition per mouse. **(G)** integrated UMAP visualization of all BM niche clusters from vehicle and ven/aza treated mice. **(H)** Stripchart representation of DE genes in each cluster between vehicle and ven/aza conditions. **(I)** Expression levels of known marker genes for different EC compartments. **(J)** UMAP visualization of miloR neighborhoods used in differential abundance testing, colored by the most abundant label they include and UMAP visualization of miloR neighborhoods colored by differential abundance testing results (log (FC)) between ven/aza and vehicle (orange: higher in ven/aza, purple: higher in vehicle). **(K)** Stripchart with differential abundance testing log (FC) results in each cluster. Each dot represents a neighbourhood. Neighbourhoods are colored with a grey-orange gradient only if their spatial FDR is below 0.05 and their absolute log (FC) is above 1.

Suppl. Fig. 2. **(A)** integrated UMAP visualization of all the BM niche cell clusters in replicate vehicle and ven/aza treated mice dataset. **(B)** Proportion plot displaying cell type composition in vehicle and ven/aza BM. **(C)** Bubble plot displaying the expression of niche factor genes such as Cxcl12, Kitl and Lepr in all stromal cell compartments captured in veh and ven/aza treated mice. **(D)** Stripchart representation of DE genes in each MSC cluster between vehicle and ven/aza conditions. **(E)** violin plots comparing the expression of Cxcl12 and Kitl in relevant stromal cell compartments between vehicle and ven/aza conditions. **(F)** Stacked violin plot of expression levels of apoptosis related genes (including Bcl2, Bcl2l11, Bcl2l1, Mcl1 and Bax) in the stroma cell clusters from vehicle control mice.

Suppl. Fig. 3. **(A)** Heatmap displaying differential gene expression for positive regulators of cell adhesion pathway in all EC clusters between ven/aza vs vehicle treated mice. **(B)** Bubble plot visualization of the expression levels of known niche factor genes typically found in ECs. **(C)** Heatmap of niche factor genes that are differentially expressed in EC clusters between ven/aza- and vehicle-treated mice. **(D)** median fluorescent intensity (MFI) of adhesion molecules Sele, Selp and VCAM in SECs from vehicle and ven/aza treated mice. Error bars indicate the S.D from 3 or 4 individual mice in each condition. Mann Whitney test was performed to determine p value between comparisons.

Suppl Fig. 4. (A) Representative flow cytometry analysis of the BM endothelial cells across all timepoints. **(B)** UMAP visualization of miloR neighborhoods colored by differential abundance testing results (log(FC)) between recovery vs ven/aza (orange: higher in ven/aza, purple: higher in vehicle) and **(C)** recovery vs vehicle. **(D)** Stripchart with differential abundance testing log(FC) results in each cluster for recovery vs ven/aza and **(E)** recovery vs vehicle. Each dot represents a neighborhood. Neighborhoods are colored with a grey-orange gradient only if their spatial FDR is below 0.05 and their absolute log(FC) is above 1. **(F)** Normalized Enrichment Score (NES) matrix for Gene Set Enrichment Analysis (GSEA) per-cluster of DE results from recovery vs vehicle-treated conditions for the EC compartment.

Suppl Fig 5. (A) Percentage of overall human CD45^{+} engraftment and percentage of overall human myeloid (CD33^{+}) and B cell lineage (CD19^{+}) engraftment. **(B)** Gating strategy to identify EPCR^{+} (CD201^{+}) HSCs, MPPs, LMPPs, CMPs, GMPs and MEPs

Supplementary Materials + Methods

Isolation and Enzymatic digestion of mouse BM cells:

The BM plug was then enzymatically digested using collagenase-type I (3 mg/ml) (C0130, Merck) and dispase II (D4693, Merck) (neutral protease, grade 2: 4 mg/ml) and incubated for 15 min at 37°C. Any residual undigested BM plug along with the flushed bones were also placed in new enzymatic digestion buffer for an additional 15 min and vortexed regularly. The digested sample was then filtered through a 100 μm strainer and resuspended phosphate saline buffer (PBS) with 2% of fetal bovine serum (FBS). The sample centrifuged at 300 g for 5 min and washed twice with PBS with 2% of FBS before immunostaining.

Irradiation

Sub-lethal irradiation was performed with a single dose at 3.75 Gy using a Cs^{137} source. Mice were culled 3 days post irradiation and isolated BM cells were characterized using flow-cytometry.

Evans Blue Dye (EBD) assay:

BM Evans Blye Dye assay was performed as per previously published protocol (1). In short, mice were intravenously injected with 25 mg dye/kg total body weight of solubilized Evans Blue Dye (Sigma-Aldrich, E2129) (solubilized in PBS). Following 3h post-injection, mice were sacrificed and cardiac perfused with 10 ml of PBS. Femurs, tibias and hip bones were collected from each mouse, cut in half and left in 300 μl of formamide (Millipore-Sigma) and incubated at 55°C overnight. Supernatant was removed the next day, and absorbance (abs) was measured at 620 nm and 740 nm. To correct for heme-containing proteins [Abs_{620} - (1.426 x Abs_{740} + 0.03)] and blanked using non-injected controls were used [corrected sample Abs_{620} —corrected non-injected control Abs_{620}].

Sample preparation and immunostaining for imaging:

Femurs from mice were collected and fixed in freshly made 4% Paraformaldehyde (PFA) (28908, Thermofisher) for 4-6 hours at 4°C. followed by PBS washing and decalcification (decalcification buffer: 0.5 M EDTA in $H_2$0, pH 7.4) for 24 hours at 4°C. Femurs were then placed in cryoprotection buffer (20% w/v sucrose (Sigma), 2% w/v polyvinylpyrrolidone (PVP) (Sigma) for a further 24 hours at 4°C and then immersed in Optimum cutting temperature (OCT) (KMA-0100-00A, CellPath) medium for 30 min on ice. Femurs were then placed in plastic caskets filled with OCT and cryo-embedded with Liquid N_2 before immunostaining.

For immunostaining, frozen slides were fixed in 99.6% cold ethanol for 15 sec before incubated in acetone for 10 min at RT. Tissues were rehydrated using S1 buffer (2) for two min before fixing in 1.6% PFA solution for 10 min at RT. Following fixation, tissue is washed 3x in S1 buffer for 5 min each and incubated in blocking buffer (mouse IgG 1 μg/ml (I53181, Sigma), rat IgG 1 μg/ml (I4131, Sigma) and sheared salmon sperm DNA 10 μg/ml (AM9680 , Thermo Fisher Scientific) in S1 buffer) in humidity chamber for 30 min. Tissue sections were washed in S1 buffer and incubated with DNA-barcode conjugated antibody mixture overnight at 4°C.

Single cell RNA-seq analysis:

Quality control and filtering:

Reads were aligned to the mouse genome reference (*mm10 3.0.0*) provided by 10X Genomics and quantified using CellRanger v. 7.1.0, aligning, in *multi*-mode. Counts were imported in R 4.3.2 into a *SingleCellExperiment* object. QC and filtering were carried out in each experimental sample (ven/aza, vehicle, recovery), discarding cells with low total number of counts (< 3.5 MADs (median absolute deviations) in log10 scale), low total number of genes with at least 1 count (< 3.5 MADs in log10 scale); high mitochondrial, ribosomal and Malat1 transcript content (> 3.5 MADs in percentage each). 1757 cells (vehicle), 2382 cells (ven/aza), and 1160 cells (recovery) were removed; 9028, 8054 and 5567 cells for vehicle, ven/aza, and recovery respectively were retained. A previously published dataset (3) with immune and stroma cells from murine bone marrow was used with *SingleR* v. 2.4.1 (4) for reference-based annotation, identifying a total of 8156 cells

as belonging to the immune system in our data (4263 vehicle, 2688 ven/aza, 1205 recovery) which were removed for further analyses.

Normalization, highly variable gene selection and dimensionality reduction:

For vehicle and ven/aza only: batch-aware normalization was performed using the *multiBatchNorm()* function from *batchelor* v. 1.18.1 (5) batching on the experimental condition, with size factors calculated via pooling using the *computeSumFactors()* function from *scran* v. 1.30.2 (6). Highly variable genes were estimated using *modelGeneVar()*, fitting a mean-variance trend to log-transformed expression values and blocking by condition. A statistical test was then performed on the variance of each gene (null hypothesis: biological component is 0). A gene is highly variable if the FDR-adjusted p-value was below 0.01, resulting in 2082 highly variable genes. Principal Component Analysis (PCA) was used to reduce the dimensionality of the log-normalized gene expression values using the highly variable genes as input features, through *runPCA()* from *scater* v. 1.30.1 (7), retaining 50 components.

Integration:

Vehicle and ven/aza samples were integrated using the Canonical Correlation Analysis-based integration procedure from *Seurat* v. 5.2.1 (8). The object was split by condition and samples were separately normalized (through *NormalizeData()*), and highly variable features were identified (through *FindVariableFeatures(), method* = *"vst"*). Datasets were integrated by running *SelectIntegrationFeatures()*, *FindIntegrationAnchors()*, and *IntegrateData()* with default parameters. Integrated counts were scaled (*ScaleData()*) and PCA was performed (*RunPCA()*) retaining the first 20 components. Finally, Uniform Manifold Approximation and Projection (UMAP) (9) was used to generate 2D embeddings using *uwot* v. 0.1.16 using the following parameters: *metric* = *"euclidean", min_dist* = *0.7, seed* = *11, n_neighbors* = *83*. Integration was diagnosed by calculating the integration Local Inverse Simpson's Index (iLISI) (10) calculated on both the un-integrated and the Seurat integrated PCA embeddings, showing a marked increase in the iLISI value towards 2.

Clustering and cell type annotation – first pass

A shared nearest-neighbor (SNN) graph was built on the Seurat-integrated PCA using the first 20 dimensions. Multi-resolution Leiden clustering was run using the *cluster_leiden()* from *igraph* v. 2.1.1(11), with resolution parameters of 0.4, 0.5, 0.6, and 0.8. The resolution of 0.6 was chosen based on the highest concordance with labels assigned through reference-based annotation, which were also used to annotate clusters. Subsequently, only clusters annotated as endothelial cells (ECs) were retained. These were re-annotated using a different reference dataset (12) through *SingleR*, resulting in the identification of one Arteriolar EC cluster, 4 Sinusoidal ECs, 2 Type R ECs, and 1 Type H EC cluster.

Symphony reference generation and projection of recovery single cell transcriptomes

Log-normalized gene expression values for the endothelial cells were used to generate a reference dataset using *buildReference()* function from *symphony* v. 0.1.1 (13). This integrates ven/aza and vehicle endothelial cells using the *harmony* algorithm (10) in a low-dimensional embedding ("corrected PCA") and a resulting UMAP. The function was run with *k = 50, umap_min_dist = 0.5, do_umap = TRUE, do_normalize = FALSE, seed = 55*. The recovery dataset was projected onto the ven/aza + vehicle reference using *mapQuery()* from *symphony*, and cell type labels were transferred using *knnPredict() symphony*, all with default parameters.

Pseudobulking and differential expression analysis

Differential expression analysis was only performed for ven/aza and vehicle data as they belong to the same experimental batch. Cells were aggregated within each cluster by summing counts across all cells belonging to the same cluster, mouse and condition using *aggregateAcrossCells()* from *scuttle* v. 1.12.0 (7), resulting in n = 3 pseudobulk replicates for vehicle clusters and n = 4 pseudobulk replicates for ven/aza clusters. The Quasi-Likelihood F test as implemented by *edgeR* (14) through *pseudobulkDGE()* from *scran* was run across all clusters between ven/aza and vehicle. FDR correction was applied within each cluster independently, and a gene is considered to be significantly differentially expressed (DE) if the FDR-corrected p-value is below 0.05. Gene Set Enrichment Analysis (GSEA) was run for each cell cluster separately using *fgsea* v. 1.28.0 (15) using genesets from the Reactome sub-collection, as included in *msigdbr* v. 7.5.1

(collection C2). Normalized Enrichment Score (NES) values for each gene set/pathway were included only if the enrichment was significant (FDR-adjusted p value < 0.05) in at least one cell cluster.

Differential abundance

Single cell transcriptomes for the three conditions (ven/aza, vehicle, and recovery) in the *symphony*-integrated space were tested for differential abundance across conditions using *miloR* v. 1.10.0 and *miloDE* v. 0.0.0.900 (16, 17). Cells were grouped into partially overlapping neighborhoods using *assign_neighborhoods*() from *miloDE* with *k = 20, order = 2, d = 20, filtering = TRUE*. These were then annotated using the cluster-based annotations through *annotateNhoods()* from *miloR*. Then, cells in each neighborhood were counted (*countCells()*) separately for each combination of individual mouse and condition, and within-neighborhood distances for each cell are defined through *calcNhoodDistance()* in the same dimensionality (*d = 20*). Differential abundance was tested (*testNhoods()*) for three contrasts: ven/aza vs vehicle, recovery vs vehicle and recovery vs ven/aza, and spatial FDR correction was applied in all cases.

Differential cell-cell interaction

Clusters in ven/aza and vehicle conditions were tested for differential cell-cell interaction using *MultiNicheNet* (18). Mouse Ligand-Receptor (LR) networks curated from Omnipath were downloaded from the NicheNet v2 Zenodo repository (DOI 10.5281/zenodo.10229221) together with the ligand-target network matrix (DOI 10.5281/zenodo.7074290), and *multinichenetr* v. 2.0.1 was used to run the *multi_nichenet_analysis()* function with the following parameters: *min_cells = 10, min_sample_prop = 0.50, fraction_cutoff = 0.05, logFC_threshold = 0.50, p_val_threshold = 0.05, p_val_adj = TRUE, top_n_target = 250, scenario = "regular", empirical_pval = FALSE, ligand_activity_down = FALSE*. The resulting output was then prioritized retrieving the top 100 interactions to draw chord diagrams, while all up-regulated interactions were considered to draw the per-cluster/per-condition violin plot.

Plotting

All plots were created using the *ggplot2* R package v. 3.5.1 (19) with the exceptions of heatmaps, which were instead created using the *ComplexHeatmap* Bioconductor package v. 2.18.0 (20,21) and the chord diagrams, which were created using the *circlize* package v. 0.4.16 (22).

1. Ramalingam P, Gutkin MC, Poulos MG, Tillery T, Doughty C, Winiarski A, et al. Restoring bone marrow niche function rejuvenates aged hematopoietic stem cells by reactivating the DNA Damage Response. Nature Communications. 2023;14(1):2018.
2. Black S, Phillips D, Hickey JW, Kennedy-Darling J, Venkataraaman VG, Samusik N, et al. CODEX multiplexed tissue imaging with DNA-conjugated antibodies. Nature Protocols. 2021;16(8):3802-35.
3. Baccin C, Al-Sabah J, Velten L, Helbling PM, Grünschläger F, Hernández-Malmierca P, et al. Combined single-cell and spatial transcriptomics reveal the molecular, cellular and spatial bone marrow niche organization. Nature Cell Biology. 2020;22(1):38-48.
4. Aran D, Looney AP, Liu L, Wu E, Fong V, Hsu A, et al. Reference-based analysis of lung single-cell sequencing reveals a transitional profibrotic macrophage. Nature Immunology. 2019; 20(2):163-72.
5. Haghverdi L, Lun ATL, Morgan MD, Marioni JC. Batch effects in single-cell RNA-sequencing data are corrected by matching mutual nearest neighbors. Nature Biotechnology. 2018; 36(5):421-7.
6. Lun AT, McCarthy DJ, Marioni JC. A step-by-step workflow for low-level analysis of single-cell RNA-seq data with Bioconductor. F1000Res. 2016; 5:2122.
7. McCarthy DJ, Campbell KR, Lun ATL, Wills QF. Scater: pre-processing, quality control, normalization and visualization of single-cell RNA-seq data in R. Bioinformatics. 2017; 33(8):1179-86.
8. McInnes LHJSNGL. UMAP: Uniform Manifold Approximation and Projection. Journal of Open Source Software. 2018.
9. Stuart T, Butler A, Hoffman P, Hafemeister C, Papalexi E, Mauck WM, 3rd, et al. Comprehensive Integration of Single-Cell Data. Cell. 2019;177(7):1888-902.e21.

10. Korsunsky I, Millard N, Fan J, Slowikowski K, Zhang F, Wei K, et al. Fast, sensitive and accurate integration of single-cell data with Harmony. Nature Methods. 2019;16(12):1289-96.

11. Csárdi G, Nepusz, T., Müller, K., Horvát, S., Traag, V., Zanini, F., & Noom, D. igraph for R: R interface of the igraph library for graph theory and network analysis (v2.1.4). Zenodo. 2025.

12. Mohanakrishnan V, Sivaraj KK, Jeong H-W, Bovay E, Dharmalingam B, Bixel MG, et al. Specialized post-arterial capillaries facilitate adult bone remodelling. Nature Cell Biology. 2024;26(12):2020-34.

13. Kang JB, Nathan A, Weinand K, Zhang F, Millard N, Rumker L, et al. Efficient and precise single-cell reference atlas mapping with Symphony. Nature Communications. 2021;12(1):5890.

14. Chen Y, Lun AT, Smyth GK. From reads to genes to pathways: differential expression analysis of RNA-Seq experiments using Rsubread and the edgeR quasi-likelihood pipeline. F1000Res. 2016; 5:1438.

15. Korotkevich G, Sukhov V, Budin N, Shpak B, Artyomov MN, Sergushichev A. Fast gene set enrichment analysis. bioRxiv. 2021:060012.

16. Dann E, Henderson NC, Teichmann SA, Morgan MD, Marioni JC. Differential abundance testing on single-cell data using k-nearest neighbor graphs. Nature Biotechnology. 2022;40(2):245-53.

17. Missarova A, Dann E, Rosen L, Satija R, Marioni J. Leveraging neighborhood representations of single-cell data to achieve sensitive DE testing with miloDE. Genome Biology. 2024;25(1):189.

18. Browaeys R, Gilis J, Sang-Aram C, De Bleser P, Hoste L, Tavernier S, et al. MultiNicheNet: a flexible framework for differential cell-cell communication analysis from multi-sample multi-condition single-cell transcriptomics data. bioRxiv. 2023:2023.06.13.544751.

19. Wickham H. ggplot2: Elegant Graphics for Data Analysis. Springer-Verlag New York. 2016.

20. Gu Z. Complex heatmap visualization. iMeta. 2022;1(3): e43.

21. Gu Z, Eils R, Schlesner M. Complex heatmaps reveal patterns and correlations in multidimensional genomic data. Bioinformatics. 2016;32(18):2847-9.

22. Gu Z, Gu L, Eils R, Schlesner M, Brors B. circlize Implements and enhances circular visualization in R. Bioinformatics. 2014;30(19):2811-2.
